# Hypothyroidism manifesting as multiple cranial neuropathies: a case report

**DOI:** 10.1186/s13256-019-2124-6

**Published:** 2019-06-13

**Authors:** Matthias Hepprich, Johannes Lorscheider, Nils Peters, Matthias Johannes Betz

**Affiliations:** 1 Department of Endocrinology, Diabetes and Metabolism, University Hospital Basel and University of Basel, Petersgraben 4, 4031 Basel, Switzerland; 2 Department of Neurology, University Hospital Basel and University of Basel, Petersgraben 4, Basel, 4031 Switzerland

**Keywords:** Hypothyroidism, Cranial neuropathy, Myxedema, Hashimoto, Myopathy

## Abstract

**Introduction:**

The clinical picture of hypothyroidism, including neurological symptoms, can be multiform, which may delay or hamper the correct diagnosis.

**Case presentation:**

We present an uncommon clinical presentation of a 38-year-old Caucasian man with mild facial palsy on the left side, uvular deviation to the left with preserved gag reflex, tongue deviation to the left, lingual dysarthria, and xerosis by severe hypothyroidism. Blood tests on admission showed elevated serum creatinine of 151 μmol/L (glomerular filtration rate 47 ml/min/1.7 CKD-EPI [Chronic Kidney Disease Epidemiology Collaboration equation]), increased creatinine phosphokinase activity (1243 U/L), markedly elevated thyroid-stimulating hormone (292.2 mIU/L), low free thyroxine level (1.1 pmol/L), and free triiodothyronine level below the limit of detection (< 0.4 pmol/L). Results of brain magnetic resonance imaging and renal ultrasound were unremarkable. Lumbar puncture revealed a normal cell count in cerebrospinal fluid, with an increased protein level of 758 mg/L and a cerebrospinal fluid/serum albumin ratio of 10.5 × 10^− 3^/L (reference range < 6.7). Further diagnostic workup did not reveal any inflammatory or infectious systemic pathologies as an underlying cause. The patient’s neurological symptoms, as well as laboratory findings including renal function, creatinine phosphokinase, and initially altered blood lipid levels, normalized with levothyroxine substitution.

**Conclusions:**

Multiple cranial neuropathy is an uncommon clinical finding in hypothyroidism, which is an important differential diagnosis in the workup of new neurological deficits.

## Background

Thyroid hormone (TH) is essential for the function and regulation of cellular metabolism in virtually all mammalian cells. Additionally, TH is of paramount importance for neurocognitive development. Signs and symptoms of severe hypothyroidism concerning the cardiovascular system or metabolism, namely bradycardia, edema, or reduced metabolic rate, are well known. Moreover, neurological complications such as lethargy and impairment of attention, as well as diffuse peripheral neuropathy and entrapment neuropathy, are common [1]. We present a case of a patient with severe hypothyroidism presenting with a rare neurological manifestation.

## Case presentation

A 38-year-old Caucasian man presented to the emergency department of our hospital with a 3-week history of dysarthria and facial weakness on the left side. A few weeks before symptom onset, he had undergone surgery for carpal tunnel syndrome on the right side. Apart from increasing fatigue and continuous weight gain of almost 20 kg over the last 2 years, his medical and family history was unremarkable. His clinical examination revealed a mild lower motor neuron facial palsy on the left, uvular deviation to the left with preserved gag reflex, tongue deviation to the left, lingual dysarthria, and xerosis. His pupils and eye movements were normal, his power and sensation for all qualities including vibration sense were preserved, his reflexes were present symmetrically, and he had no ataxia. Facial fullness and edematous extremities were noted. His mucous membranes were unremarkable, but his skin was dry. His vital parameters were normal apart from an increased body mass index (31.6 kg/m^2^) and hypothermic tympanic temperature of 35.6 °C (blood pressure 127/79 mmHg, heart rate 70 beats/min).

His blood test results on admission showed elevated serum creatinine of 151 μmol/L (glomerular filtration rate 47 ml/min/1.7 CKD-EPI [Chronic Kidney Disease Epidemiology Collaboration equation]) and increased creatinine phosphokinase (CK) activity (1243 U/L). Results of brain magnetic resonance imaging (MRI) and renal ultrasound were unremarkable. Cerebrospinal fluid (CSF) analysis revealed a normal cell count but increased protein levels of 758 mg/L and a CSF/serum albumin ratio of 10.5 × 10^− 3^/L (reference range < 6.7) without signs of intrathecal immunoglobulin production or oligoclonal bands. Results of serological testing were unremarkable. MRI of the brain did not show any pathologic lesions or contrast enhancement, especially within the cranial nerves. Multiple cranial neuropathy was presumed, and the patient was admitted to the department of neurology.

On the following day, routinely performed thyroid function tests detected markedly elevated levels of thyroid-stimulating hormone (TSH) (292.2 mIU/L), low free thyroxine levels (1.1 pmol/L), and free triiodothyronine levels below the limit of detection (< 0.4 pmol/L). A diagnosis of overt hypothyroidism was made, and levothyroxine therapy in a dose of 100 μg/d (1.0 μg/kg body weight, 1.3 μg per ideal body weight) was started after hypocortisolism was excluded. Ultrasound revealed a small thyroid gland (4.8 ml) with an inhomogeneous pattern but without signs of focal lesions. Further diagnostic workup showed a markedly reduced basal metabolic rate on indirect calorimetry (1380 kcal/d, reference 1950 kcal/d) and elevated antibodies against thyroid peroxidase (495 IU/mL, reference range < 34 IU/mL). When the patient was discharged 1 week after admission, his symptoms were unimproved. Six weeks later, the patient was seen in our outpatient clinic and reported a substantial improvement of speech quality and general well-being. On clinical examination, no residual pathologic signs were observed. The patient’s levothyroxine dosage was eventually increased to 150 μg/d, and his levels of TSH and free T4 normalized (Table 1). At the following visit, 5 months after initiation of levothyroxine substitution, the patient’s facial features had changed substantially, and all his neurologic symptoms had completely resolved. A loss of 2 kg body weight was noted. The remainder of his physical examination was unremarkable. Laboratory analysis showed normal levels of thyroid hormones and TSH as well as normal creatinine phosphokinase and kidney parameters. Increased total and low-density cholesterol levels at initial presentation also had normalized at the 5-month follow-up visit.

**Table 1:** Laboratory results at initial presentation and follow-up visits (in bold values below or above reference)

	Initial presentation	6 Weeks later	5 Months later	Reference range
Levothyroxine dosage, μg/d	–	100.00	150.00	
Leukocytes, 10^9^/L	6.06	6.67	7.80	3.5–10.0
Hemoglobin, g/L	148.00	138.00	144.00	140–180
Creatinine, μmol/L	**151.00**	**99.00**	91.00	49–97
Urea, mmol/L	**7.90**	6.40	5.00	3.2–7.3
ASAT, U/L	**59.00**	26.00	nd	11–34
ALAT, U/L	33.00	31.00	nd	9–59
LDH, U/L	**417.00**	203.00	nd	135–225
Creatinine phosphokinase (CK), U/L	**1243.00**	106.00	nd	50–200
CK-MB, μg/L	**18.40**	nd	nd	< 5.0 μg/L
Triglycerides, mmol/L	1.57	1.74	0.72	< 1.7
Total cholesterol, mmol/L	**6.71**	3.18	4.05	3.0–5.2
HDL cholesterol, mmol/L	1.63	0.85	1.37	0.9–2.2
LDL cholesterol, mmol/L	**4.37**	1.54	2.35	16.0–3.40
Thyrotropin (TSH), mIU/L	**292.00**	2.69	2.19	0.332–4.490
Free T4, pmol/L	**1.10**	21.00	19.30	11.6–22.0
Free T3, pmol/L	**< 0.4**	5.50	5.40	2.6–5.6
Total T4, nmol/L	**6.00**	nd	nd	66–181
Total T3, nmol/L	**< 0.3**	nd	nd	1.2–3.2
Cortisol, nmol/L	449.00	nd	nd	80–638
Glucose, mmol/L	5.00	nd	4.50	3.8–6.1
Glycated hemoglobin A1c, %	5.30	nd	nd	4.8–5.9

*Abbreviations: ALAT* Alanine aminotransferase, *ASAT* Aspartate aminotransferase, *CK-MB* Muscle/brain creatine kinase, *HDL* High-density lipoprotein, *LDH* Lactate dehydrogenase, *LDL* Low-density lipoprotein, *nd* Not determined, *T3* Triiodothyronine, *T4* Thyroxine, TSH Thyroid-stimulating hormone

## Discussion

We report an unusual case of severe hypothyroidism due to autoimmune thyroiditis with myxedema, neuromyopathy, and renal affection. Hypothyroidism is the leading thyroid disorder worldwide, with autoimmune causation being the most common [2], and a prevalence of 3.05% in Europe with a female preponderance [3]. Typical signs of hypothyroidism such as fatigue, weight gain, hypothermia, dry skin, and myxedema with facial fullness, as in our patient, are well known but are neither sensitive nor specific, and the clinical picture can vary substantially with a slow long-term progression often leading to non- or misdiagnosis [2].

Thyroid hormones are ubiquitously required for development and maintenance of cellular functions and are paramount for the development of the central nervous system [4]. Thus, thyroid hormone deficiency can affect the central and peripheral nervous systems. A decline or slowing of neurocognitive functions (memory loss, ataxia), probably due to altered synaptic plasticity, is commonly observed in patients with myxedema, but it is often only partially reversible even under appropriate treatment [2].

In our patient, the clinical syndrome of multiple clinical neuropathy without clinical evidence of further involvement of the peripheral nervous system led to the initial differential diagnosis of an inflammatory cranial neuropathy or a brainstem pathology. However, MRI showed no lesions in the brainstem or inflammatory changes of the cranial nerves. CSF protein levels were increased, which is most likely related to blood-brain barrier dysfunction rather than an autoimmune process in patients with overt hypothyroidism and has been reported to be fully reversible [5]. While diffuse peripheral neuropathy and entrapment neuropathy are common in hypothyroidism—the latter being found in up to 35% of patients—dysfunction of cranial nerves is seen much less frequently [1, 6]. Compression of the optic chiasm by pituitary gland enlargement, as well as hearing impairment, may occur and can be detected already in an early stage of the disease with visual and brainstem auditory evoked potentials [1, 7]. However, to the best of our knowledge, multiple cranial neuropathy with facial and lower cranial nerve palsy has not been reported previously. In general, neuropathy in hypothyroidism seems to be caused primarily by a metabolic dysfunction due to the lack of thyroid hormone and thereby disrupting the function of the myelin sheath rather than a compressive neuropathy due to mucinous deposits [8].

Muscular manifestations such as weakness, cramps, and myalgia are common in hypothyroidism but can be oligosymptomatic, as in our patient, despite markedly elevated CK levels at initial presentation.

Renal impairment in hypothyroidism is associated with increase of serum creatinine and decline of glomerular filtration rate. It is caused by direct effects on glomerular and tubular function and indirectly through alterations in cardiac and vascular function, as well as decreased renin-angiotensin system leading to lowered renal plasma flow [9]. In turn, after levothyroxine substitution, renal function improves significantly or normalizes fully [9, 10], as seen in our patient.

The long duration with initial hypothyroid symptoms starting already 2 years before cranial nerve symptoms occurred, with extremely low thyroid hormone levels at presentation, may be an explanation for the clinical symptoms of our patient. Most of the patients may present at earlier stages before onset of symptoms. When untreated, severe hypothyroidism can be life-threatening and can lead to myxedema coma. This is usually precipitated by an additional hit, such as an acute infection, trauma, or other forms of stress [11]. Shortly before his initial presentation, our patient underwent surgery for carpal tunnel syndrome, which may have been prevented or could have led to an earlier diagnosis of the underlying thyroid disorder.

## Conclusions

As hypothyroidism can cause a wide variety of neurological symptoms, any unclear neurological findings should prompt thyroid function testing. Multiple cranial neuropathy is a rare manifestation of hypothyroidism and was fully reversible in our patient with levothyroxine substitution.

## Abbreviations

ALAT: Alanine aminotransferase
ASAT: Aspartate aminotransferase
CK-MB: Muscle/brain creatine kinase
CSF: Cerebrospinal fluid
HDL: High-density lipoprotein
LDH: Lactate dehydrogenase
LDL: Low-density lipoprotein
MRI: Magnetic resonance imaging
T3: Triiodothyronine
T4: Thyroxine
TH: Thyroid hormone
TSH: Thyroid-stimulating hormone
